# Levodopa medication improves incidental sequence learning in Parkinson's disease

**DOI:** 10.1016/j.neuropsychologia.2016.09.019

**Published:** 2016-12

**Authors:** M. Beigi, L. Wilkinson, F. Gobet, A. Parton, M. Jahanshahi

**Affiliations:** a Sobell Department of Motor Neuroscience and Movement Disorders, UCL Institute of Neurology, Queen Square, London WC1N 3BG, UK; b Division of Psychology, Department of Life Sciences, Brunel University, Uxbridge UB8 3PH, UK; c Behavioral Neurology Unit, National Institute for Neurological Disorders and Stroke, 10 Center Drive, Bethesda, MD, United States; d Department of Psychological Sciences, University of Liverpool, Liverpool L69 7ZA, UK

**Keywords:** Parkinson's disease, Incidental sequence learning, Serial reaction time, Levodopa medication, Striatum, Basal ganglia

## Abstract

Empirical evidence suggests that levodopa medication used to treat the motor symptoms of Parkinson's disease (PD) may either improve, impair or not affect specific cognitive processes. This evidence led to the ‘dopamine overdose’ hypothesis that levodopa medication impairs performance on cognitive tasks if they recruit fronto-striatal circuits which are not yet dopamine-depleted in early PD and as a result the medication leads to an excess of dopamine. This hypothesis has been supported for various learning tasks including conditional associative learning, reversal learning, classification learning and intentional deterministic sequence learning, on all of which PD patients demonstrated significantly worse performance when tested on relative to off dopamine medication. Incidental sequence learning is impaired in PD, but how such learning is affected by dopaminergic therapy remains undetermined. The aim of the current study was to investigate the effect of dopaminergic medication on incidental sequence learning in PD. We used a probabilistic serial reaction time task (SRTT), a sequence learning paradigm considered to make the sequence less apparent and more likely to be learned incidentally rather than intentionally. We compared learning by the same group of PD patients (*n*=15) on two separate occasions following oral administration of levodopa medication (on state) and after overnight withdrawal of medication (off state). Our results demonstrate for the first time that levodopa medication enhances incidental learning of a probabilistic sequence on the serial reaction time task in PD. However, neither group significantly differed from performance of a control group without a neurological disease, which indicates the importance of within group comparisons for identifying deficits. Levodopa medication enhanced incidental learning by patients with PD on a probabilistic sequence learning paradigm even though the patients were not aware of the existence of the sequence or their acquired knowledge. The results suggest a role in acquiring incidental motor sequence learning for dorsal striatal areas strongly affected by dopamine depletion in early PD.

## Highlights

• Implicit sequence learning is impaired in non-medicated Parkinson's Patients.
• l-Dopa medication ameliorates implicit sequence learning deficits.
• Patients showed no indications of awareness of sequence structure.
• Such deficits can be identified with greater sensitivity using a within groups comparison.

## 1 Introduction

Levodopa medication has been described as the most significant advance in the treatment of Parkinson's disease (PD) (Olanow et al., 2004, Poewe et al., 2010, Stocchi, 2005). By ameliorating the effects of dopamine depletion in the basal ganglia, levodopa can improve the major motor symptoms with considerable benefits for patients’ quality of life. Nonetheless, the consequent widespread increase in dopamine levels can impair functions in brain networks that are relatively spared in the early stages of the disease, such as the limbic and orbitofrontal striatal circuits. This ‘dopamine overdose’ can result in cognitive deficits that have a negative impact on the quality of life of patients with the disease (Schrag et al., 2000). Fundamental processes such as learning and memory have been linked to dopamine release, especially within the pre-frontal cortex and striatum (see for example Gabrieli, 1998; Packard and Knowlton, 2002; Shohamy et al., 2008; Williams and Goldman-Rakic, 1995). Therefore, studying such effects has potentially important implications for the management of PD as well as increasing our theoretical understanding of the role of dopamine in modulating neural pathways instantiating cognition and learning.

Incidental procedural learning is the gradual acquisition of a cognitive or motor skill in long-term memory through repetitive task performance (Nissen and Bullemer, 1987, Reed and Johnson, 1994, Shanks et al., 2003). In contrast, intentional declarative learning involves the active acquisition of factual knowledge. Incidental motor learning has been widely investigated using the serial reaction time task (SRTT). Typically, in this task, participants respond to the appearance of a target at one of several locations by pressing the corresponding response button without knowing that the targets follow a pre-determined sequence (Nissen and Bullemer, 1987). Learning of the sequence is demonstrated by the speeding up of reaction times (RTs) on sequence relative to random (or pseudorandom) trials. Many researchers argue that such learning can occur in the absence of awareness due to the existence of good incidental learning in normal participants who cannot demonstrate explicit awareness of the sequence structure (Berns et al., 1997; Cleeremans et al., 1998; but see also Shanks et al. (2003), Shanks (2004)) and the presence of relatively preserved incidental learning in patients with intentional learning deficits, such as Korsakoff's syndrome (Nissen and Bullemer, 1987) and Alzheimer's (Knopman and Nissen, 1987). Nonetheless, researchers concede that parallel mechanisms of intentional (explicit) learning may be engaged in SRTTs if the sequence structure is insufficiently concealed (Rowland and Shanks, 2006; Shanks et al., 2005).

Interest in incidental motor sequence learning in patients with PD has been fuelled by studies in normal participants that both identify task-related activity in brain networks known to be affected by PD and suggest a significant role for dopamine in task performance. Functional imaging studies of incidental SRTT in healthy participants relate performance to change in activation within the basal ganglia, especially the caudate and putamen (Grafton et al., 1995, Rauch et al., 1997, Schendan et al., 2003, Willingham et al., 2002), and to cortical regions associated with the fronto-striatal network, including the pre and supplementary motor areas (SMA) (Grafton et al., 1995, Hazeltine et al., 1997, Honda et al., 1998) and, during early stages of learning, the dorsolateral prefrontal cortex (DLPFC) (Meehan, et al., 2011). Furthermore, a systematic release of dopamine in the left putamen and bilaterally in the anterior caudate during SRTT performance has been documented using PET to measure changes in the concentration of the dopamine receptor ligand ^11^C-raclopride (Badgaiyan et al., 2007). Learning was attributed to activity in the left caudate as it was not found in a matched motor planning task, whilst the activity common to both tasks (left putamen and right caudate) was proposed to underlie movement selection. Finally, the administration of raclopride (a D2 antagonist) produces impairment in learning proportional to the dose administered (Tremblay et al., 2009). Taken together, these studies indicate that learning on the SRTT relies on the striatal motor and associative circuits known to be adversely affected by dopamine depletion in PD, and that successful learning appears to be related to a systematic release of dopamine. Nonetheless, as the relation of dopamine to performance is described by an inverted U curve, where too little or much dopamine may be detrimental to performance, it is unclear the degree to which patients with PD will be impaired on SRTT learning on or off medication (Cools et al., 2001, Goldman-Rakic, 1999, Williams and Goldman-Rakic, 1995).

Several studies have investigated performance of the SRTT in patients with PD on levodopa therapy. The majority reported impaired learning compared to age matched controls (e.g. Brown et al., 2003; Jackson et al., 1995; Kelly et al., 2004; Muslimovic et al., 2007; Shin and Ivry, 2003; Wilkinson et al., 2009), but a few studies found little difference between PD and control groups (Doyon et al., 1997, Feigin et al., 2003). Nonetheless, a meta-analysis of SRTT studies in PD patients taking l-Dopa concluded that learning was impaired (Siegert et al., 2006). However, it is not possible to differentiate, on the basis of these studies, the degree to which impairments were a consequence of the disease or its medical treatment with dopamine replacement therapy.

Only two studies have specifically reported data on SRTT learning in PD patients who were either not taking levodopa (Muslimovic et al., 2007) or tested off-medication after a washout period (Wilkinson and Jahanshahi, 2007). In the first study, Muslimovic and colleagues (2007) assessed learning in a large sample of PD patients (n=95) performing a 10 item SRTT. The patients displayed some sequence learning but this was attenuated in comparison to healthy age matched controls. Yet, the learning for a subgroup of ‘de novo’ patients (n=24) who were not receiving dopamine therapy was indistinguishable from aged matched participants. However, in examining a sub-group of ‘de novo’ patients who did not require levodopa as yet, the effects of medication are inextricably confounded with disease stage and severity in this study. This is demonstrated by the non-medicated patients being significantly more recently diagnosed and less impaired (on both the Hoehn and Yahr and UPDRS scales) compared to the remaining patients. Furthermore, on the SRTT the non-medicated patients had similar overall RTs to control participants whereas medicated patients were significantly slower. In the second study, Wilkinson and Jahanshahi (2007) demonstrated that SRTT learning in PD patients (treated with dopaminergic medication) tested *off* dopaminergic medication was impaired compared with healthy age matched controls. Hence, this suggests that impaired learning on the SRTT can occur as a function of the disease and not simply as a side-effect of the medication. However, patients were only tested off-medication and comparisons of the magnitude of the effect with published studies are not really possible due to large inter-individual variability in the presentation of the disease and the considerable differences in task procedures across studies. As a consequence, the relationship between impairments in PD patients on and off dopamine medication remains undetermined.

The current study aims to address the effects of PD and levodopa medication on incidental motor sequence learning by assessing the same group of patients both on and off dopamine medication. As learning on the task is likely to be mediated by both the motor and associative fronto-striatal circuits, it was predicted that similar to the motor symptoms of PD, sequence learning would be greater on medication compared to the off state. Importantly, the study uses an SRTT in which the sequence is presented probabilistically, which is considered ideal for minimising explicit knowledge of the sequence and ensure the sequence learning remains implicit (Cleeremans and McClelland, 1991, Stadler, 1992).

## 2 Methods

### 2.1 Participants

Fifteen individuals (13 male; 14 right handed and one ambidextrous) meeting the Parkinson's Disease Society Brain Bank diagnostic criteria for idiopathic PD (Hughes et al., 1992) gave written informed consent to participate, which included the willingness to be assessed prior to taking medication following an overnight washout period (see below). They received reimbursement for travel expenses. Ethics approval for the study was obtained from the Joint Ethics Committee of the UCL Institute of Neurology & the National Hospital for Neurology and Neurosurgery. Informed consent was obtained from all participants. Participants were aged between 54 and 75 (*M*=67.1, *SD*=6.1), had been diagnosed for between 3 and 21 years (*M*=8.9, *SD*=5.3) and were classified by a neurologist as being in the mild to moderate stages of the disease, i.e. with Hoehn & Yahr (1967) scores between 1 and 3 (*M*=1.8, *SD*=.7). Severity of motor symptoms was assessed using the motor section of the Unified Parkinson's Disease Rating Scale (UPDRS, part III Fahn & Elton, 2005) with scores ranging between 12 and 62 (*M*=30.8, *SD*=13.8) off medication and between 5 and 39 (*M*=15.0, *SD*=8.8) on medication. Participants were non-demented with mean scores on the Mini-Mental State Examination (Folstein, Folstein, & McHugh, 1975) of 29.4 (*SD*=.7) and non-depressed with average scores of 7.43 (SD=3.7) on the Beck Depression Inventory (Beck, et al. 1961). Pre-morbid IQ was estimated using the National Adult Reading test (Nelson and O'Donnell, 1978). All participants were taking levodopa (Sinemet, Madopar or Staleva) and the mean levodopa equivalent daily dose (LEDD) was 694.89 (*SD*=462.44) milligrams (Tomlinson et al., 2010). Information about the patients is presented in Table 1. Patient data was compared to that of thirteen control participants (5 male, all right handed, mean age=55.1 SD=10.1) with no history of neurological or psychiatric illness, head injury or alcohol or drug abuse who were also tested on two occasions to control for potential practice effects.

### 2.2 Design

In a repeated measures design, all participants learned two parallel probabilistic sequences in separate test sessions, one on and the other off medication, with their order counterbalanced and at least one week apart. The mean time off medication before testing was 11.33 h (SD=1.6, ranging from 9 to 14 h withdrawal).

### 2.3 Procedure

#### 2.3.1 Apparatus and probabilistic serial reaction time task

**Fig. 1:**
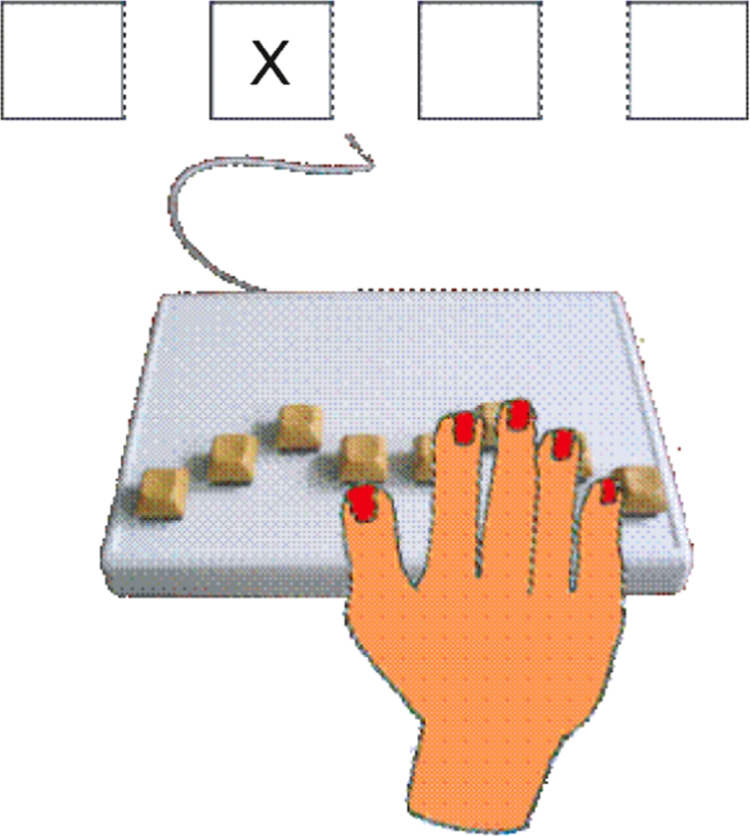
Illustration of the button box used with an example of the stimuli presented on the monitor.

Stimulus presentation, response recording and RT measurement were performed using a Dell laptop PC with a 15.1″ TFT monitor and an ergonomically designed button box (see Fig. 1). The four response buttons were arranged horizontally (and are labelled as 1–4 from left to right).

Participants performed a four-choice SRTT comprising 15 blocks of 100 trials. The beginning of each trial was signalled by the appearance of a black 'X' (17 mm) in the centre of one of four boxes (26 mm square with a black outline and white background), which were evenly spaced (17 mm separation) across the horizontal mid-line of the grey display. Participants were instructed to respond to the location of the target by pressing the corresponding button on the response box as quickly and accurately as possible. Their response triggered the removal of the target cross and its appearance (after 400 ms) at the next location. At the end of each block participants were required to press a button when they were ready to continue.

Target locations in each session were selected from one of two pairs of second order conditional sequences (SOCS) in which determining the current location requires as a minimum knowledge of the previous two locations. (Pair 1 is SOC1=3-1-4-3-2-4-2-1-3-4-1-2 and SOC2=4-3-1-2-4-1-3-2-1-4-2–3, pair 2 is SOC3=1–2-1-4-3-2-4-1-3-4-2-3 and SOC 4=3-2-3-4-1-2-4-3-1-4-2-1.) Sequences were created according to rules proposed by Reed and Johnston (1994). Half of the participants in each session (on vs. off medication) were randomly assigned either to pair 1 or 2 and received the other pair in their next session. In each testing session, the SOC sequence participants were expected to learn was presented with an 85% probability and so is referred to as the probable SOC. The other paired SOC was presented on the remaining 15% of trials, and is referred to as the improbable SOC. The next location was selected from the relevant SOC using the same probabilistic rule (i.e. two most recent target locations). For example, for a participant trained on SOC1, the locations 4 & 1 were followed by either a target at location 2 (following the *probable* sequence SOC1) with a probability of.85 or a target at location 3 (following the *improbable* paired sequence SOC2) with a probability of.15. Each block began at a random point in the sequence and the SOCs used as probable or improbable trials were counterbalanced across participants.

Reaction times (RT) were measured in milliseconds from the onset of the target to initiation of a response. For each participant, median RTs and errors for both probable and improbable trials in each block were calculated. In the analysis, RTs were included for both correct and incorrect responses, as a large proportion of errors, especially off-medication, are likely due to simple difficulties in kinetic control rather than incorrect response selection, and allows comparison with previously published studies (see Wilkinson and Jahanshahi (2007)).

#### 2.3.2 Tests of awareness

At the end of the second testing session following completion of the SRTT task, awareness of the sequence was assessed by two sets of tests: the process dissociation procedure and a recognition test. These involve informing the participant of the presence of the sequence and so could not be performed in session 1 without revealing the nature of the study. The process dissociation procedure (PDP) determines whether the generation of knowledge of the probable sequence was under intentional control (and therefore conscious). Following the final SRTT block of learning in session 2, participants were informed that during the training blocks the targets had appeared in a repeating sequence. For 12 of the 24 test trials participants observed short test sequences of five targets taken from the probable sequence to which they responded as they had previously. Then, they were instructed to press a button indicating the next item in the sequence (inclusion test, I). For the remaining 12 trials participants had to produce a single key press that did not overlap with the probable sequence (exclusion test, E). The recognition test involved identifying chunks from the training sequence. Before the test, participants were told they would be presented with short sequences of 6 target locations some of which were part of the training sequence and some were not. Participants were requested to respond to each target, judge whether the sequence was old or new and rate of how confident they were in their judgement. Ratings were made by clicking on option buttons labelled “old” and “new” and then on buttons labelled “sure,” “fairly sure,” and “guess.”

## 3 Results

### 3.1 Probabilistic sequence learning

**Fig. 2:**
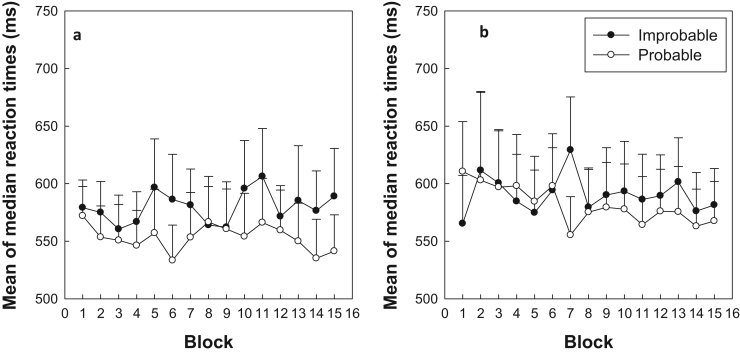
a and b: Mean of the median reaction times in milliseconds (ms) for probable and improbable trials, plotted separately for patients with Parkinson's disease on (2a) and off (2b) levodopa medication across 15 blocks of the Serial RT task. Error bars represent one standard error of the mean.

Fig. 2 shows mean of the median RTs for patients on (Fig. 2a) and off (Fig. 2b) dopaminergic medication across 15 blocks of trials. A four-way ANOVA was performed on median RTs with Medication (on vs. off), Probability (probable vs. improbable trials), and Block (1–15) as within subjects variables and Order (session 1 vs. session2). As the results showed that neither the main effect nor any interactions involving order were significant (P>0.1) the analysis was repeated as a three-way ANOVA excluding this factor. Participants were slower on improbable than probable conditions (Probability: *F*(1,14)=16.77, *p*<.001), which indicates the presence of learning. There were also significant interactions between Medication x Probability: *F*(1,14)=4.9, *(p*=.044), indicating that the extent of learning (i.e. difference between improbable and probable trials) differed significantly between medication states. Additionally, there was a significant interaction between Probability x Block: *F*(14,196)=2.89, (*p*=.001), which is indicative of a generalised increase in learning across blocks. No other main effects or interactions were significant (p>.10).

**Fig. 3:**
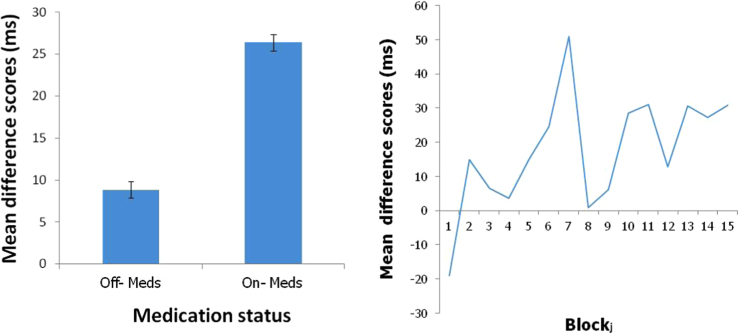
a and b. Mean RT difference scores (improbable minus probable reaction times) plotted (a) as an overall mean across blocks for each of the two medication conditions and (b) for blocks 1–15 collapsed across medication conditions.

To fully explore the two significant interactions a direct measure of learning was derived by calculating the mean differences between probable and improbable scores. To illustrate the basis for the Medication x Probability interaction the mean difference score in the two medication states are plotted in Fig. 3a, which clearly shows that learning was far higher when patients were tested in the on medication condition. To illustrate the Probability x Block interaction difference score were calculated on a block-by-block basis and averaged across the two medication conditions (see Fig. 3b). The Page trend test was then used to assess whether learning tended to increase over the blocks using the large sample adjustment (Page, 1963). The results revealed a significant trend for increased learning across blocks (L=10.9, p<.001).

The patient data was then compared to controls separately for the on and off medication conditions in separate ANOVAs. However, it should be noted that the primary motivation of this study was a repeated measures patient design with an independent variable (medication) that cannot easily be tested in controls and is further complicated by being counterbalanced across the testing sessions. Specifically, the timing of the medication conditions was counterbalanced so that half of the patients received medication at testing time 1 and the other half at time 2 and so each group comprised patients tested at the two times (i.e. testing time is a between group variable). A control comparison group was created by randomly selecting 50% of the participants and using their data gathered at time 1 with the remainder of the control group comprising the time 2 data from the other patients. Patients and controls were compared using a four factor mixed ANOVA with Probability and Block as within participant factors and Group (Patient vs. Control) and test order (time 1 vs time 2) as between participant factors. In both cases learning was confirmed by a main effect of probability when comparing controls with patients On- (F(1, 24)=20.18, p<.001) and Off- (F(1, 24)=8.01, p=.008) medication. Furthermore, the development of this learning over time is demonstrated by the Probability x Block interaction in both On- (F(14, 336)=1.77, p=.044) and Off- (F(14, 336)=3.54, p<.001) comparisons. In neither case did patients differ significantly from controls, On- (F(1, 24)=1.68, p=.208) and Off- (F(1, 24)=2.43, p=.132) medication. There were no other significant main effects or interactions. We also repeated the analysis using the reverse grouping of control data (e.g. creating a group with the time 1 and time 2 data not included in the original analysis). The analysis produced exactly the same pattern of significant and non-significant results. Additionally, we also analysed the whole control sample using a three way ANOVA (with Probability, Block and Order as factors) to see if there were any indications of order effects. There was no main effect of Order nor any interactions between Probability and Order (alone or in combination with Block).

A three-way ANOVA with Medication (on vs. off), Probability (probable vs. improbable trials) and Block (1−15) as the within subject factors was performed on mean percentage errors and revealed neither significant main effects nor any interactions (p>.1).

### 3.2 Tests of awareness

#### 3.2.1 Process dissociation procedure (PDP)

**Fig. 4:**
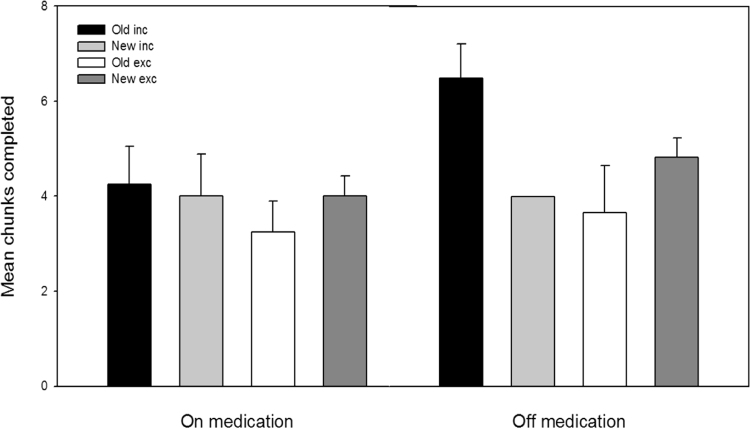
Mean number of test chunks completed with either the final item of a triplet from the trained sequence (old) or an untrained sequence (new) on and off levodopa medication. Completions were calculated out of a possible 12 that could have been achieved in the inclusion and exclusion tests. Error bars represent one standard error of the mean.

Task performance in the inclusion (I) and exclusion (E) tests was scored by calculating the number of sequences that were completed with an item from the probable (trained) SOC (Wilkinson and Jahanshahi, 2007). A significant difference between the inclusion and exclusions score would indicate that participants had control over the expression or withholding of their knowledge, providing performance in either condition was not at chance. To estimate baseline performance, the number of sequences that were completed with an item from the improbable SOC were calculated for both tests. Fig. 4 shows the number of probable versus improbable sequence completions in the inclusion and exclusion tests for each medication condition. The data was analysed using a three-way ANOVA with Task (inclusion vs. exclusion), Sequence (probable vs. improbable) and Medication (on vs. off) as a within groups variable. No main effects or interactions were significant (Task, *F*(1,12)=2.411), Sequence, *F*(1,12)=.261, Medication, *F*(1,12)=.103, Task x Medication, *F*(1,12)=.151, Sequence x Medication, *F*(1,12)=1.265, Task x Sequence, *F*(1,13)=.305, Task x Sequence x Medication, *F*(1,13)=.128. Participants were equally likely to select items from the probable SOC whether they were trying to complete the sequence under inclusion conditions or deliberately trying to avoid probable items under exclusion instructions. They also completed sequences using a similar number of items from the improbable sequence (e.g. responses were equally distributed). Therefore, we conclude the PDP shows no evidence that participants had developed awareness of the sequences.

#### 3.2.2 Recognition test

**Fig. 5:**
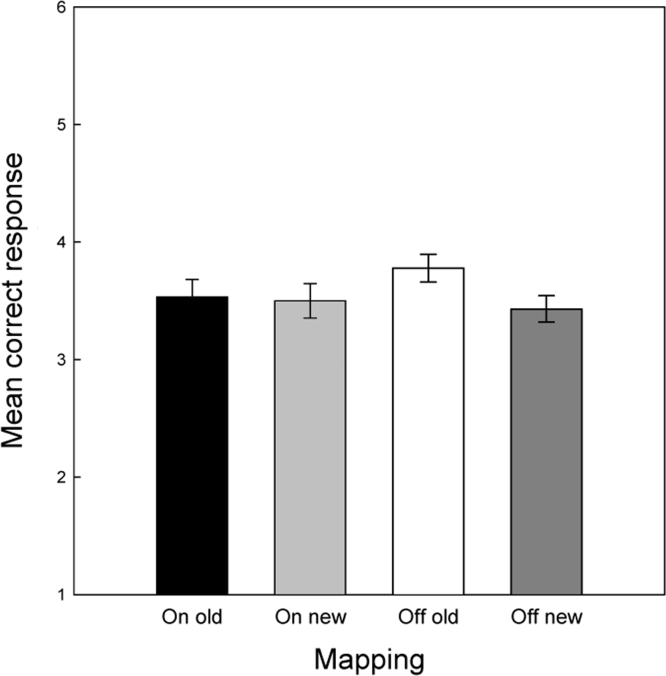
Mean recognition ratings for old and new test sequences on and off levodopa medication. Participants responded to 12 old and 12 new sequences and made a recognition judgement for each sequence (1=certain new, 6=certain old).

Responses and certainty judgements on the recognition test were classified according to the following criteria 1= certain new, 2= fairly certain new, 3=guess new, 4=guess old, 5=fairly certain old and 6= certain old. Separate mean scores were calculated for probable and improbable sequences with the expectation that probable sequences were more likely to be judged as old (previously encountered sequences) than improbable sequences. A two-way ANOVA with sequence (probable vs. improbable) as the within subject variables and Medication (on vs. off) as the between groups variable revealed no significant effects of either Sequence (*F*(1,12)=.526) or Medication, (*F*(1,12)=1.456) nor any interaction between (*F*(1,12)=1.015) them, and so there is no evidence of explicit sequence knowledge (Fig. 5).

## 4 Discussion

Patients with Parkinson's disease showed significantly greater incidental sequence learning on an incidental probabilistic SRTT while tested on dopaminergic medication than when assessed off medication. This finding was, in large part, attributable to a marked attenuation of learning in the off-medication condition. Crucially, there was no evidence for any significant overall difference in RTs between the medication conditions, which makes it unlikely that differences in learning on and off medication can be simply explained by greater motor difficulties when off medication. Furthermore, as indicated by the absence of main effects of block, there was no evidence for performance improvements based simply on practice or task familiarity effects. Importantly, in all conditions there was an interaction between block and probability, which was attributable to a trend for increased learning with time in all conditions. As a consequence, the results clearly indicate that the administration of levodopa to PD patients can, at least in part, ameliorate deficits in incidental sequence learning associated with the disease.

In general when we compared patient performance to normal control data we found no evidence of impairment on- or off- medication. In part, this supports the rationale of this study that the effects of the disease versus those of medication is best understood using a within participant design, which has greater sensitivity within a heterogeneous patient group. Though, as noted here comparing such studies to control data can be challenging as certain conditions cannot reasonably replicated (e.g. medication).

These findings add to mounting evidence regarding the importance of dopamine during incidental sequence learning tasks, such as the SRTT (Badgaiyan et al., 2007, Jackson et al., 1995). The current study extends earlier results by demonstrating that considerable attenuation, but not complete abolition, of PD patients’ ability to learn incidental motor sequences can occur as a consequence of the disease. This result was shown through the highly significant difference between patients on and off medication, but the failure of either of these groups to differ from controls indicates the importance of within group comparisons in understanding the effects of medication. Interestingly, the opposite pattern of results were reported in a study looking at *intentional* motor sequence learning using an SRTT task (Kwak et al., 2010). In their task patients on medication were specifically impaired during early trials where learning was being established compared with either the same group off-medication or the performance of age-matched control participants. However, participants were informed they would be learning short (6-item) motor sequences, and were even shown the sequence in a preview period before training. In contrast, in the current study participants were not informed of the presence of the sequence and showed no objective evidence of awareness of the sequence structure at the end of training (likely due to the more complex sequence structure and probabilistic presentation).

The current result is consistent with previous studies of probabilistic sequence learning in PD that reported no evidence of awareness of the sequence (Wilkinson and Jahanshahi, 2007, Wilkinson et al., 2009). Importantly, task awareness might also account for differences between the current study and previous studies of motor sequence learning in which PD patients were explicitly instructed and aware they were to learn a sequence. For example, a series of studies have investigated the impact of levodopa (Carbon et al., 2003, Feigin et al., 2003, Ghilardi et al., 2007) on sequence learning in a reaching paradigm in which a cursor is moved to a sequence of 8 target locations. The results indicated that explicit learning of the target sequences during testing was no different for the on vs. off medication conditions, but that declarative knowledge was greater in the dopamine depleted state (Feigin et al., 2003, Ghilardi et al., 2007). In the task used in these studies, PD patients would have engaged mechanisms for intentional and goal-directed learning, which may differ from the incidental learning of sequences in the present study. This would also account for the differences between the current study and the SRTT study of Kwak et al. (2010). Taken together, the results across these studies indicate the heterogeneity of motor learning tasks in their dependence upon brain regions where dopamine is depleted in PD. As a consequence, care should be taken when generalising findings, e.g. suggesting that results apply across all motor sequence learning when only incidental learning has been assessed.

In general, the degeneration of dopamine production in PD follows a distinctive spatial-temporal gradient with the greatest loss of DA neurons in early stages occurring in the lateral ventral tier of substantia nigra pars compacta (SNpc) that enervates the dorsal striatum (Fearnley and Lees, 1991, Kish et al., 1988). In contrast, in early PD, dopamine activity is relatively preserved in ventral striatal areas and the cortical areas to which they are interconnected, that is the anterior cingulate, inferior temporal cortex and the orbitofrontal cortex (OFC). Of particular relevance to the current study are reviews of the literature arguing that differential effects of dopaminergic medication within the dorsal (improved by l-dopa administration) and ventral striatum (impaired by an ‘overdose’ of l-dopa medication) may have specific implications for learning (Cools, 2006, Macdonald and Monchi, 2011). In terms of motor learning, it has been argued that skill acquisition is critically dependent on the ventral striatum and skill performance on the dorsal striatum (Atallah et al., 2007). More recently, MacDonald et al. (2011, 2013) have refined this dichotomy suggesting that the ventral striatum mediates memory encoding (for both explicit and implicit tasks) whilst the dorsal stratum is involved in memory retrieval once a skill is learned.

The current study, however, indicates that dysfunction of the dorsal striatum as in PD also has implications for motor skill learning. By providing a continuous measure of learning throughout the task (e.g. learning as indexed by RT differences between probable and improbable sequences across trials), the current paradigm is ideal for identifying difficulties in acquisition as opposed to retrieval of well-established learning. The absence of a consistent difference between the probable and improbable sequence trials across all blocks in the off medication condition is strong evidence that patients are failing to learn the probable sequence in this off state. This contrasts with paradigms that test learning at the end of the practice period which may be testing the retrieval of established learning. Additionally, the similar RTs between patients on and off medication provide further evidence that impairments are not attributable to a retrieval difficulty. Nonetheless, even though patients were more greatly impaired off medication this does not mean that they were unimpaired when on medication. Indeed, Wilkinson et al. (2009) have previously shown using the same probabilistic learning task that PD patients on medication demonstrate less learning than age matched controls. Therefore, the results do not preclude the involvement of the ventral striatum in sequence learning.

In conclusion, the present study established for the first time that levodopa medication enhanced incidental learning by patients with PD on a probabilistic sequence learning paradigm even though the patients were not aware of the existence of the sequence or their acquired knowledge. The results suggest a role in incidental motor sequence learning for dorsal striatal areas strongly affected by dopamine depletion in early PD.

## Funding

This work was funded by the ESRC (R84003) and Parkinson’s UK.

## Figures and Tables

**Table 1:** Demographic and clinical characteristics of Parkinson's disease patients who took part in the study. SD=standard deviation, UPDRS-III: motor section of the Unified Parkinson's Disease Rating Scale. NB: Data marked with an asterisk was unavailable for participant 15.

***PD Patients (n=15)***	***Mean***	***SD***
*Age (years)*	*67.07*	*6.06*
Disease duration (years)*	*8.86*	*5.25*
UPDR-III ON*	*15.00*	*8.75*
UPDRS-III OFF*	*30.79*	*13.81*
*Levodopa equivalent daily dosage*	694.89	462.44
*Length of Medication Withdrawal (Hours)*	*12.88*	*2.20*
*Hoehn and Yahr stage of illness*	*1.75*	*.67*
National Adult Reading Test Predicted IQ*	*92.83*	*8.39*
*Mini Mental State Examination (range 0–30)*	*29.43*	*.76*
Beck Depression Inventory (range 0–63)*	*7.43*	*3.65*
