# Supplementary material for: Levodopa medication improves incidental sequence learning in Parkinson's disease
Source: Neuropsychologia. 2016 Dec;93(Pt A):53–60. doi: 10.1016/j.neuropsychologia.2016.09.019 (PMC5155668; doi:10.1016/j.neuropsychologia.2016.09.019)
Supplement: Supplementary file 1 — Supplementary material [file mmc1.docx]

**Supplementary Material – S1**

a

c

b

**Figures S1:** Mean of the median reaction times in milliseconds (ms) for probable and improbable trials, plotted separately for patients with Parkinson’s disease on (S1a) and off (2b) levodopa medication and (2c) controls across 15 blocks of the Serial RT task. Error bars represent one standard error of the mean. Neither medication group differed significantly from the controls nor were there any significant interactions between group and other factors. However, the relatively large error bars highlight the difficulty of such between group comparisions, and reinforce the rationale of our study of looking at difference by within group comaprisions.
